# Sleep Disorders and Dual Disorders

**DOI:** 10.1192/j.eurpsy.2022.1183

**Published:** 2022-09-01

**Authors:** L. Fernandez Mayo, D. Baño Rodrigo, E. Barbero García, M. Agujetas Rodriguez, I. Falcón Torres, V. De Antonio Pérez, C. Medina Sanchez, M. Serrano García

**Affiliations:** CAID Majadahonda, Addictions, Madrid, Spain

**Keywords:** Cannabis, dual disorder, alcohol, sleeping disorders

## Abstract

**Introduction:**

While it is well known that there is an interaction between sleep disorders and substance abuse, it is certainly more complex than was previously thought. The effects on sleep depend on the substance used, but it has been shown that both during use and in withdrawal periods consumers have various sleep problems, and basically more fragmented sleep. We know that sleep problems must be taken into account to prevent addiction relapses.

**Objectives:**

To explain the different sleep disorders caused by substances such as alcohol and cannabis

**Methods:**

As an example of this, two cases are introduced: the first one, a 17-year-old boy, who is diagnosed with ADHD with daily cannabis use since the age of 14. As a result of reducing consumption, he presents an episode of sleep paralysis that he had not previously had. The second one is a 50-year-old man diagnosed with a personality disorder and with dependence on cannabis and alcohol for years. He currently has abstinence from alcohol for months and maintains daily cannabis use. However, he has long-standing sleep pattern disturbances and frequent depersonalization phenomena at night.

**Results:**

Alcohol at low doses has no clear effects on sleep architecture. At higher doses it decreases sleep latency, as well as awakenings. In chronic alcoholic patients, a decrease in deep slow sleep, and more fragmented sleep have been found. Cannabis withdrawal reduces sleep quality, increases latency, and produces strange dreams.

**Conclusions:**

There is a positive relationship both between having a substance use disorder and suffering from a sleep disorder.

**Disclosure:**

No significant relationships.

